# Antibiotic resistance of *Escherichia coli* from the milk of Ettawa crossbred dairy goats in Blitar Regency, East Java, Indonesia

**DOI:** 10.14202/vetworld.2023.168-174

**Published:** 2023-01-27

**Authors:** Tweedekharis Marlin Agatha, Prima Ayu Wibawati, Reza Ikhza Izulhaq, Bodhi Agustono, Ragil Angga Prastiya, Dhandy Koesoemo Wardhana, Abzal Abdramanov, Widya Paramita Lokapirnasari, Mirni Lamid

**Affiliations:** 1 Department of Veterinary Medicine, School of Health and Life Sciences (SIKIA), Universitas Airlangga, Surabaya 60115, Indonesia; 2 Department of Veterinary Science, Division of Veterinary Public Health, Faculty of Veterinary Medicine, Universitas Airlangga, Surabaya 60115, Indonesia; 3 Department of Veterinary Sanitary Expertise and Hygiene, Faculty of Veterinary medicine, Kazakh National Agrarian Research University, Almaty, Kazakhstan; 4 Department of Veterinary Science, Division of Animal Husbandry, Faculty of Veterinary Medicine, Universitas Airlangga, Surabaya 60115, Indonesia

**Keywords:** antibiotic drugs, *Escherichia coli*, Indonesia, public health, specific gravity

## Abstract

**Background and Aim:**

Antimicrobial resistance, especially antibiotic resistance, is one of the most severe public health challenges. Antibiotic resistance occurs when bacteria avoid and fight the mechanism of action of antibiotic drugs. This study aimed to determine the resistance of *Escherichia coli* from the milk of Ettawa crossbreed dairy goat at Blitar Regency, East Java, Indonesia, with the antibiotics streptomycin, sulfonamides, and trimethoprim.

**Materials and Methods:**

A total of 34 milk samples of Ettawa crossbreed dairy goats were used in this study. The initial stages of this research included tests of the physical properties, isolation, and identification of *E. coli*. Then, the *E. coli* isolates were tested for antibiotic resistance using the Kirby–Bauer method.

**Results:**

The results showed that all samples were positive for *E*. *coli*. The physical properties of milk, namely, color, odor, flavor, and consistency, were normal. The results of the alcohol test showed normal acidity, and the specific gravity of goat milk met the criteria, with an average specific gravity of 1.0295 g/mL. The results of the antibiotic resistance test showed that 4 (12%) samples were resistant to streptomycin, 5 (15%) to sulfonamide, and 3% to trimethoprim.

**Conclusion:**

The prevalence of *E. coli* from Ettawa crossbreed dairy goats in Blitar Regency, East Java, Indonesia, was 100%. Furthermore, this *E. coli* isolate exhibited resistance to antibiotics streptomycin, sulfonamides, and trimethoprim. The use of antibiotics in the dairy goat industry in Indonesia should be controlled to prevent the spread of resistant *E. coli* from animals to humans through the food chain and prevent the emergence of multidrug-resistant *E. coli*.

## Introduction

Antibiotic-resistant pathogenic bacteria pose a serious public health challenge worldwide. Resistance to antimicrobial agents has become a significant source of morbidity and mortality worldwide**,** with 4.95 million deaths associated with complications from resistant bacterial infections [1]. Antibiotic resistance occurs when bacteria can avoid and defeat the drugs designed to kill them [2]. Antimicrobial resistance (AMR) is associated with the use and abuse of antibiotics in various fields, one of which is animal husbandry [3]; therefore, it is necessary to conduct a health assessment in animal husbandry because human health is closely related to animal health [4]. Antimicrobials in intensive animal production systems are frequently used to maintain livestock health, welfare, and productivity. An increase in the demand for animal protein has led to an over-reliance on antimicrobials because their use promotes growth [5] and antimicrobials are administered to animals with no diagnosed illness [6].

The challenges of AMR faced by Indonesia are similar to those of many other developing countries that misuse and overuse antibiotics in humans, livestock, and aquaculture [7]. Interviews with goat breeders in Blitar Regency, East Java, Indonesia, revealed that streptomycin, sulfonamides, and trimethoprim are the most commonly used antibiotics for goats. The use of antibiotics aims to overcome the problem of digestive disorders that sometimes occur in some livestock. Antibiotics commonly used in animal husbandry are influential in developing pathogenicity and resistance in bacteria. Bacteria will increase due to resistance that can cause infectious diseases. This bacterial resistance makes the treatment of infectious diseases no longer effective. Antibiotics can cause loss of material, life, quality, etc., and lead to the failure of health programs [8].

*Escherichia coli* is an indicator of sanitation and hygiene [9, 10]. According to Ercumen *et al*. [11], *E. coli* contamination occurs through animal feces. *Escherichia coli* is pathogenic in humans because several serotypes can produce toxins. These bacteria are one of the causative agents of mastitis in cattle. The incidence of mastitis in dairy cattle can decrease the quality and quantity of milk produced [12]. The presence of *E. coli* contamination in milk and related samples (cow nipples, milking hands, milking equipment, and cow dung) has been reported and milk samples have recorded the highest percentage of *E. coli* contamination (68.0%) [13]. A high level of *E. coli* was reported in ducks and duck-related samples, with an average occurrence of 78.00% [14].

This study aimed to determine the presence of antibiotic-resistant *E. coli* in Ettawa crossbreed dairy goat milk to ensure public health.

## Materials and Methods

### Ethical approval

Ethical approval for animal research was not required as live animals were not used in this study. Milk samples were purchased from the farmer of dairy goats. Milk samples from Ettawa Crossbreed goats, with healthy conditions during lactation, produce at least 1 L/day of milk. Milking is done by hand directly in the morning (06.00–06.30 am). Samples of fresh milk taken as much as 100 mL. Each sample was placed in a sterile milk bottle. Each bottle was labeled and stored in an ice box (4°C) and, then transported to the laboratory.

### Study period and location

This study was conducted from March to May 2022. The milk samples were collected from the Alastika Jaya dairy goat farm, Gandusari District, Blitar Regency, East Java, Indonesia. Identification of *E. coli* and its antimicrobial resistance test were carried out at the Satwa Sehat Laboratory, Malang and SIKIA Banyuwangi, Universitas Airlangga.

### Organoleptic tests

Testing included color, odor, flavor, and consistency tests [15]. The color test was performed by placing 5 mL of milk in a test tube and then looking at the background of the test tube on white paper to observe any color abnormalities. The odor test on milk was performed by placing 5 mL of milk in a test tube and then smelling it. Milk easily absorbs the odor around it due to the fat that it contains. A flavor test was performed by pouring some milk on the palm and evaluating the change in taste. For the taste test, the milk was brought to a boil first to ensure hygiene. For the consistency test, 5 mL of milk was put in a test tube and shaken slowly. Residual oscillations on the walls of the test tube were observed. If it ends speedily or disappears, then the milk was considered diluted.

### Alcohol test

An alcohol test was conducted to test the degree of acidity in milk and to observe the reaction of raw goat milk with ethyl alcohol. The alcohol test was performed by adding 5 mL of 70% alcohol to 5 mL of milk and observing the presence of lumps in the milk [16]. The alcohol test was declared positive if lumps or granules were formed. If no granules were formed, the test was negative.

### Determination of specific gravity of milk

The specific gravity test was performed by stirring the milk thoroughly and pouring it into a 100 mL cylinder. Then, the lactodesimeter (Funke-Gerber^®^) was carefully dipped in the milk such that it floats. During the reading, care was taken to ensure that the lactodesimeter did not touch the inner surface of the cylinder to avoid any error. Then, measurements were made and the indicated scale was read. The lactometer shows only the second and third decimals of the specific gravity but does not show 1.0. For example, a reading of 28 means the specific gravity equals 1.028 [17].

### Isolation and identification of *E. coli*

Isolation and identification of *E. coli* were performed by plating bacteria on Mac Conkey’s agar (MCA, HiMedia, India), eosin methylene blue agar (EMBA, HiMedia), Gram staining, and identification by biochemical tests, namely, IMViC test, consisting of indole production test, methyl red (MR) test, Voges-Proskauer (VP) test, and citrate test [18].

#### Isolation of bacteria on MCA and EMBA media

Bacteria were isolated according to the National Standardization Agency of Indonesia [17]. Samples were plated on MCA media (HiMedia) and incubated at 37°C for 24 h. After the colonies suspected of being *E. coli* appeared, they were cultured on EMBA media (HiMedia) at 35°C ± 0.5°C for 24 h. *Escherichia coli* colonies are metallic green, with a black dot in the middle.

#### Gram-stain

The smear preparation for suspected *E. coli* was made on an object glass that had been cleaned using 70% alcohol, followed by a fixation on a Bunsen flame, dipping in crystal violet (Sigma-Aldrich, USA), and waiting for 1–2 min. After settling, the dye was removed and aerated. After drying, the isolate was dipped in an iodine solution, allowed to stand for 1–2 min, rinsed with running water, and dipped in acetone alcohol. Then, the sample was washed with running water and dried. A few drops of safranin (Sigma-Aldrich) were added to the sample. The sample was allowed to stand for 40 s, rinsed, and dried. The shape of the suspected *E. coli* cells was observed using a microscope. Bacteria are classified as Gram-positive if the cells appear purple and Gram-negative if they are red [17].

#### Identification of isolates by biochemical tests

Bacteria were identified using several tests [18]; (a) Indole test, suspected colonies from each EMBA were transferred into tryptone water ((Merck, Germany; 1.10859.0500) for the indole test and incubated (24 ± 2 h at 35°C). After incubation, 2–3 drops of Kovach reagent were applied. The test results were positive for the presence of a red ring on the surface of the media. (b) The MRVP test was performed by inoculating suspected *E. coli* isolates and incubating them for 24 h. After incubation, 3–5 drops of MR were added to the samples. Then, the samples were homogenized and incubated for ±4 h (37°C). Positive results were marked in red and negative in yellow. The VP test was performed by plating suspected *E. coli* colonies on VP media (HiMedia) followed by incubation at (35°C ± 0.5°C, 48 h). After the media turned cloudy, Barrit’s reagent, which consisted of a solution of 0.2 mL 40% KOH in sterile distilled water and 0.6 mL of 5% naphthol in ethanol, was added. This was followed by the addition of the VP reagent. Positive results showed a change in color from yellow to dark red. (c) For the citrate test, suspected *E. coli* colonies were plated on Simmons’ citrate agar (HiMedia) and incubated for 96 h at 35°C. A positive reaction was indicated by the presence of blue color, whereas an adverse reaction was indicated by green [17, 18]. (d) The motility test was performed by inoculating suspected *E. coli* isolates to sulfide indole motility media and incubated at 37°C for 24 h. Positive results are indicated by the presence of a white root-like distribution around the inoculation.

### Resistance test

The Kirby–Bauer disk diffusion method was used to evaluate antibiotic resistance in *E. coli*. This method used Mueller–Hinton agar (MHA, Merck) with the pour method to determine the diameter of antibiotic resistance. This method produces qualitative categories with sensitive, intermediate, and resistant assessments based on the standard Clinical and Laboratory Standards Institute (CLSI) (Table-1) [19].

**Table-1:** Interpretation standard of diameter in inhibition zone [19].

Antibiotic	Disk Concentration (µg)	Sensitive (mm)	Intermediate (mm)	Resistant (mm)
Streptomycin	10	15	12–14	11
Sulfonamides	250	17	13–16	12
Trimethoprim	5	15	11–15	10

A 0.2 mL suspension of bacteria was put in a Petri dish containing MHA (Merck) and, then leveled with a bent glass rod so that the bacteria stick to the medium. The bacteria were allowed to stand for 10–15 min. The antibiotic (Oxoid, UK) on the disk was placed on the surface, and then the plate was incubated at 37°C for 24 h. The diameter of the resulting inhibition zone was measured with a caliper and then compared with the standard from CLSI.

### Statistical analysis

The data were presented descriptively in percentages displayed in tables.

## Results

### Physical properties of milk

The results of the color, odor, flavor, consistency tests, alcohol test, and specific gravity test for goat milk were normal (Table-2) [15, 18].

**Table-2:** Physical properties test of goat milk.

Item test	Result
Color	Yellowish-white color*
Odor	Fresh goat’s milk*
Taste	Savory taste*
Viscosity	Watery consistency*
Alcohol test	Sediment in a fine or small shape (normal acidity degree)*
Specific gravity (g/mL)	1.0295*

* In accordance with standards [15, 18]

### Prevalence of *E. coli* in goat milk

These results indicate the percentage of *E. coli* found in the milk of the Ettawa crossbreed dairy goat at Blitar Regency, East Java, Indonesia. Thirty-four milk samples (100%) were positive for *E. coli*. The results of the identification test *E. coli* are summarized in Table-3.

**Table-3:** The results of the identification test *Escherichia coli* in Ettawa crossbreed dairy goats milk at Blitar Regency.

Morphology	Gram-stain	Biochemical test					Result (n=34)

		INDOL	MR	VP	SCA	SIM	
Short rods and short chains, Solitary	Gram-negative	+	-	+	+	Motile, Indole+, H2S-	*Escherichia coli*

### Prevalence of antibiotic resistance of *E. coli* in goat milk

Of the 34 milk samples, antibiotic resistance to streptomycin was 12%, sulfonamide was 15%, and trimethoprim 3%. The results of *E. coli* resistance to antibiotics are summarized in Table-4.

**Table-4:** The results of the number of samples on the antibiotic resistance test of streptomycin, sulfonamide, and trimethoprim against bacteria *Escherichia coli*.

Antibiotic	Laboratory result		

	Sensitive (%)	Intermediate (%)	Resistant (%)
Streptomycin	29 (85)	1 (3)	4 (12)
Sulfonamides	15 (44)	14 (41)	5 (15)
Trimethoprim	26 (76)	7 (21)	1 (3)

## Discussion

The quality of raw goat milk fit for consumption should normal, clean, white or cream color, natural flavor without any foreign matter and adulteration; furthermore, in the alcohol test, the sediment should be fine or small and specific gravity should be between 1.028 and 1.034 at 20°C [15]. The results of the physical properties of the Ettawa crossbreed’s milk sample met the above criteria, with an average specific gravity of 1.0295 (Table-2). The white color of the milk results from the scattering of visible light by casein micelles and fat globules. In contrast, the yellowish color of milk is caused by the presence of fat-soluble substances, such as carotene, from plant sources in the diet [20].

In atmosphere dominated by silage or animal odors, volatile compounds may be transferred directly from the surrounding environment to the milk before, during, and after milking. Moreover, the odor of the male goat in rut is often a source of the goaty flavor in fresh goat milk. The flavor of milk from each animal is different based on genetic and physiological characteristics, feeding systems and diets, and environmental conditions [21]. Previous studies have reported that differences in goat breeds affect the content of long- and medium-chain fatty acids and potentially contribute to the unique aroma and flavor of milk [22, 23].

Milk viscosity is one of the important benchmarks of milk quality. Normal milk that is good for consumption is runny or liquid and does not clot. These tests are performed in accordance with the Indonesian National Standard [17]. The viscosity of milk is influenced by its composition, concentration, pH, and temperature [20, 24, 25].

Specific gravity, the mass of a certain quantity of material divided by its volume, is dependent on the temperature at the time of measurement, the temperature history of the material, composition of the material (especially the fat content), and air (a complication with more viscous products). With all of this in mind, the specific gravity of milk varies from 1.027 to 1.033 g/mL at 20°C [26]. The higher the specific gravity of the milk, the better it is, because the milk content becomes more concentrated, water content is low, and the percentage of non-fat ingredients increases. In contrast, more fat in milk lowers the specific gravity of milk. The specific gravity of milk varies somewhat with breed [20] and temperature (i.e., as temperature increases and specific gravity decreases) [27].

Food-borne diseases in public health programs prioritize the surveillance of milk food-borne diseases by monitoring food-borne pathogens and microbial contamination in milk products. Hence, dairy farms are compromised to reduce the milk contamination source from the udder and the dairy cattle’s health status and the production environment through improved hygiene practices in cattle management and milk handling [28].

*Escherichia coli* is widely found in the digestive tract and is often found in environments with poor sanitation [29]. In this study, high *E. coli* contamination was found in 34 samples (100%) of Ettawa crossbreed dairy goat milk (Table-3). In other studies, *E. coli* contamination was reported in 9.3% (7/75) raw goat milk samples in Luxor Governorate, Egypt [30] and 40% (100/400) samples in Taif province, Saudi Arabia [31]. This bacterium can be transmitted from various sources, one of which is water [32]. The water supply in northern Tanzania may be a source of AMR *E. coli*.

*Escherichia coli* is a Gram-negative commensal bacterium, commonly used as an indicator during surveillance and monitoring for antibiotic resistance [33]. *Escherichia coli* was tested in this study because it is commonly found in dairy products and is often found in conjugated plasmids that are commonly transferred between enteric bacteria [34]. They often find new ways to develop resistance and can sometimes share these abilities with other bacteria, increasing the spread of resistance; for example, *E. coli* ST131, quickly spread in the community and among healthcare settings. These strains often cause more severe infections and spread more easily [35] and have a great capacity to accumulate resistance genes, primarily through horizontal gene transfer [36]. *Escherichia coli* of animal origin often show resistance to mostly older antimicrobial agents, including tetracyclines, phenicols, sulfonamides, trimethoprim, and fosfomycin [37]. In a study, 55 *E. coli* isolates from ducks in Penang, Malaysia, were resistant (100%) to vancomycin, 92.7% to tetracycline, 72.7% to ampicillin, 67.3% to streptomycin, and 67.3% to sulfamethoxazole-trimethoprim [38]. This is in line with the findings of Adzitey *et al*. [39], who reported 50% resistance to amoxicillin, trimethoprim, and tetracycline in *E. coli*.

This study was conducted to determine food safety using three types of antibiotics, namely, streptomycin, sulfonamide, and trimethoprim, used by farmers to treat infections caused by *E. coli*. The study revealed that *E. coli* is resistant to three antibiotics. In the 34 samples studied, several samples were resistant to these antibiotics (Table-4). Thus, the public must increase vigilance, considering that bacteria resistant to antibiotics easily transmit their resistance to other bacteria [40]. A recent study reported a substantial increase in global antibiotic consumption in 79 countries between 2000 and 2015 and predicted a further 200% increase by 2030 [41]. The presence of antimicrobial-resistant *E. coli* in the broiler farm environment, with ESBL-producing isolates of SHV-12 type, has been reported [42]. For *E. coli*, resistance to third-generation cephalosporins and combined resistance to third-generation cephalosporins, fluoroquinolones, and aminoglycosides increased significantly at the EU/EEA level between 2013 and 2016 [43].

Streptomycin is widely used in the treatment of microbial infections, with the primary mechanism of action being inhibition of translation by binding to the ribosome. Moreover, streptomycin interacts with MscL and activates it, thus leading to an outward flux of K^+^, thereby increasing the potency of the drug [44]. Sulfonamides, used to treat various Gram-positive and Gram-negative bacterial strains [45] and protozoal infections, act as structural analogs of para-aminobenzoic acid, inhibiting dihydropteroate synthase competitively [46]. Sulfonamide drugs are commonly used in veterinary medicine and serve as antibacterial compounds to treat livestock diseases, such as gastrointestinal and respiratory tract infections [47]. In line with this study, Li *et al*. [48] found that sulfonamide-resistant *E. coli* (*sul3* positive in *E. coli*) *sul3* is a more recent version of the gene associated with sulfonamide resistance.

Trimethoprim is widely used to treat *E. coli* infections; however, its efficacy is limited given the rapidity with which trimethoprim resistance develops [49] through mutations in the gene encoding the trimethoprim target [50].

The discovery of *E. coli* resistance to several antibiotics in Ettawa crossbreed goat milk that is consumed by the community must be accompanied by vigilance so that resistance does not spread more widely. Some resistance mechanisms emerge but do not develop after the first explosion, but others can spread around the world very quickly [51]. This is because microorganisms can develop resistance to the drugs used, and most pathogenic organisms can develop resistance to at least some antimicrobial agents through mechanisms of resistance (limiting uptake of a drug, modification of a drug target, inactivation of a drug, and active efflux of a drug) [52].

## Conclusion

In this study, we found AMR to *E. coli* (100%) in milk from Ettawa crossbreed dairy goat in Blitar Regency, East Java, Indonesia. Antibiotic resistance to streptomycin, sulfonamide, and trimethoprim was 12%, 15%, and 3%, respectively. The presence of *E. coli* in goat milk for human consumption can cause milk-borne diseases, and the discovery of microbial resistance can seriously impact public health. The incidence of microbial resistance leads to prolongation of treatment and recovery.

## Authors’ Contributions

TMA, PAW, and ML: Conceptualization and design of the study. TMA, BA, RAP, and RII: Collected samples. TMA, PAW, RII, and DKW: Performed the laboratory procedures. BA, RAP, AA, and WPL: Analyzed and interpreted the data. PAW and ML: Writing-original draft. DKW and AA: Writing-review and editing. WPL: Edited the manuscript. All authors have read and approved the final manuscript.
